# Patterned Neuroprotection in the *Inpp4a^wbl^* Mutant Mouse Cerebellum Correlates with the Expression of *Eaat4*

**DOI:** 10.1371/journal.pone.0008270

**Published:** 2009-12-14

**Authors:** Andrew J. Sachs, Samuel A. David, Neena B. Haider, Arne M. Nystuen

**Affiliations:** 1 Department of Genetics, Cell Biology and Anatomy, University of Nebraska Medical Center, Omaha, Nebraska, United States of America; 2 Department of Ophthalmology and Visual Sciences, University of Nebraska Medical Center, Omaha, Nebraska, United States of America; Baylor College of Medicine, United States of America

## Abstract

The *weeble* mutant mouse has a frame shift mutation in inositol polyphosphate 4-phosphatase type I (*Inpp4a*). The phenotype is characterized by an early onset cerebellar ataxia and neurodegeneration, especially apparent in the Purkinje cells. Purkinje cell loss is a common pathological finding in many human and mouse ataxic disorders. Here we show that in the *Inpp4a^wbl^* mutant, Purkinje cells are lost in a specific temporal and spatial pattern. Loss occurs early in postnatal development; however, prior to the appearance of climbing fibers in the developing molecular layer, the mutant has a normal complement of Purkinje cells and they are properly positioned. Degeneration and reactive gliosis are present at postnatal day 5 and progress rapidly in a defined pattern of patches; however, *Inpp4a* is expressed uniformly across Purkinje cells. In late stage mutants, patches of surviving Purkinje cells appear remarkably normal with the exception that the climbing fibers have been excessively eliminated. Surviving Purkinje cells express *Eaat4*, a glutamate transporter that is differentially expressed in subsets of Purkinje cells during development and into adult stages. Prior to Purkinje cell loss, reactive gliosis and dendritic atrophy can be seen in *Eaat4* negative stripes. Our data suggest that Purkinje cell loss in the *Inpp4a^wbl^* mutant is due to glutamate excitotoxicity initiated by the climbing fiber, and that *Eaat4* may exert a protective effect.

## Introduction

The *weeble* (*Inpp4a^wbl^*) mouse mutation spontaneously arose on a C57BL/6J-A^w^ background. Affected mice are characterized by severe cerebellar ataxia and failure to thrive, visibly noticeable by postnatal day 9 (P9). Extensive neurodegeneration occurs in the cerebellum, especially evident in Purkinje cells, and CA1 pyramidal neurons of the hippocampus. The ataxia is presumably due to Purkinje cell dysfunction and loss. The degenerative phenotype in the *Inpp4a^wbl^* mutant is unique in that Purkinje cells degenerate relatively early compared to other ataxic mouse mutants, yet there is no prenatal developmental abnormality. The mutant phenotype is caused by a single base deletion in the gene inositol polyphosphate 4-phosphatase type I (*Inpp4a*) [1]. This gene is highly expressed in Purkinje cells and to a much lesser degree throughout the brain. The enzyme catalyzes the removal of the 4-position phosphate from inositol 3,4-bisphosphate (Ins(3,4)P_2_), inositol 1,3,4-triphosphate (Ins(1,3,4)P_3_), and phosphatydlinositol 3,4-bisphosphate (PtdIns(3,4)P_2_) [2].

In the cerebellar Purkinje cell, an excitatory glutamate signal from the climbing fiber is transduced through metabotropic glutamate receptor 1 (GRM1) [3], [4]. This causes the generation of inositol 1,4,5-triphosphate (IP_3_) via guanine nucleotide binding protein, alpha q polypeptide (GαQ) [5] and phospholipase C beta 4 (PLCβ4) [6]. IP_3_ binds IP_3_ receptor 1 (ITPR1) and causes the release of Ca^2+^ [7], [8]. Then a series of enzymes add and subtract phosphate groups to breakdown IP_3_ to Ins. Six early onset ataxic mouse mutants have been identified that have mutations in genes from this system including: *Grm1^tm1Crpl^* [9], [10], *Gnαq^tm1Soff^* [11], *Plcβ4^tm1Hssh^* [6], *Itpr1^opt^* [12], [13], *Inpp4a^wbl^* [1] and *Car8^wdl^* [14]. These genes are expressed at high levels in Purkinje cells; however, only the *Inpp4a^wbl^* mutant is degenerative.

Purkinje cell degeneration is a noted feature of several neurological disorders in the mouse and human. Histologically, Purkinje cells appear as a uniform monolayer of cells; however, gene expression studies have shown that Purkinje cells differentially express genes in distinct compartments. Genes such as, *Aldoc*, *Plcβ4*, *Ebf2* and *Eaat4* demarcate subdivisions of the Purkinje cell population into complex patterns of parasagittal stripes [15]–[19]. These patterns are extended to climbing fiber projections from the inferior olive [20], [21]. The functional consequences of these subdivisions are not completely understood, but they are reflected in certain phenotypes, which may indicate that they influence disease progression [22]–[25]. For example, in the mouse mutant *tottering*, Purkinje cells that do not express *Aldoc* are preferentially lost [26], while the opposite is true in the *nervous* mutant [27]. Defining these patterns of Purkinje cell loss will likely lead to the identification of protective genes and suggest disease mechanisms at the molecular level.

In this report, we show that perinatal Purkinje cell degeneration in the *Inpp4a^wbl^* mutant follows a specific pattern. The onset of cell loss coincides with modeling of climbing fibers from the inferior olive and Purkinje cell dendritic arbors in the developing molecular layer. The pattern of loss is not defined by the expression of the mutant *Inpp4a* allele, but is correlated to the expression of *Eaat4*. Interestingly, Purkinje cells in protected *Eaat4* positive parasagittal stripes lose climbing fiber input in late stage mutants, which has not been documented before in other mutants. These data suggest not only the probable initiating factor and mechanism for cell death, but also a potential protective strategy.

## Methods

### Ethics Statement

All animals were bred and maintained under standard conditions at The University of Nebraska Medical Center research vivarium in accordance with a protocol approved by the Animal Care and Use Committee at the University of Nebraska Medical Center. Mice were housed in microisolator cages and provided food and water ad libitum. The University of Nebraska Medical Center is in compliance with the NIH policy on the use of animals in research (Animal Welfare Act P.L. 89–544, as amended by P.L. 91–579 and P.L. 94–279) as well as the Guide for the Care and Use of Laboratory Animals, NIH Publication No. 86–23. A mating cross of heterozygous BALB/cJ^wbl/+^ was established to generate mutant and control mice.

### Genotyping

DNA was isolated from tail tips excised from pups using a standard protocol [1]. Genotyping for the *Inpp4a^wbl^* mutation was performed using a multiplex PCR reaction with allele specific primers: wtF 5′CTACGGATCCTGGCGCAGA, wtR 5′CTGTCGTAGGCACGTGCTCA, mutF 5′AGTGTCAGGCACTGCTTGGA, mutR 5′GAGGGACGCTCTCTGACATT, resulting in the following bands: wild type, 135 bp; mutant, 277 bp. DNA was amplified by PCR using the following conditions: 40 ng of DNA in a 10 µl PCR reaction mixture containing 1.25 µl PCR buffer (100 mM Tris-HCl [pH 8.8], 500 mM KCl, 15 mM MgCl_2_, and 0.01% w/v gelatin), 200 µM each dATP, dCTP, dGTP, and dTTP, 2.5 pmol of each forward and reverse primer, 0.25 U Taq polymerase. Reaction mixtures were subjected to 40 cycles of 94°C for 30 s, 55°C for 30 s and 72°C for 30 s. Products were electrophoresed on a 4% agarose gel containing ethidium bromide, and visualized by UV transillumination.

### Phenotypic Analysis

Age matched adult heterozygous and wild-type mice were trained for three consecutive days. The rotorod was set to a constant minimum speed of 4 rpm. Mice were placed on the rotorod for 5 minutes, then given 5 minutes of rest. This was repeated twice more for three runs per day. Testing was performed one week after training. The rotorod was set to accelerate from 4 rpm to 40 rpm over a period of 5 minutes. Times were recorded from the start of acceleration until a mouse fell off the apparatus or clung to rod for a full rotation. Testing consisted of three trials per mouse with 15 minutes between trials. Lifespan was measured from postnatal day 0 to the time of first visible distress when the mutants were euthanized.

### Immunohistochemistry for Paraffin Embedded Tissues

Tissues were dissected from euthanized mutant and age matched littermates and fixed in either 4% PFA in PBS or methanol:acetic acid(3∶1). Tissues were paraffin embedded in either sagittal or coronal planes and serially sectioned at 10 µm thick. Three mutant and control mice were taken from each timepoint. Sections were mounted on Poly-L-Lysine (Sigma) coated slides. Immunohistochemistry was performed as before (Sachs et al., 2007) using antibodies specific to CALB (Swant), VGLUT2 (Chemicon), PLCβ4 (BD Biosciences), INPP4A (Santa Cruz), GFAP (Chemicon), EAAT4 (Santa Cruz). Slides were treated in the following washes to deparaffinize and hydrate: xylene, 10 min, two changes; 100% ethanol, 5 min, two changes; 95% ethanol 5 min, 70% ethanol 3 min, PBST (Tween 0.1%) 5 min. PFA fixed section were boiled in 10 mM sodium citrate (pH 6.5) and allowed to cool to room temperature. Tissue sections were blocked in PBST, 5% horse serum for 1 hour; then incubated with primary antibody in blocking solution overnight at 4°C. Primary antibody solution was washed off with 3 changes of PBST for 5 minutes each and then incubated with secondary antibody (AlexaFluor 488 or 555, Invitrogen) 1∶200 in blocking solution. Slides were washed in 3 changes of PBST for 5 min each and briefly counterstained with DAPI, washed in PBS and mounted with Vectashield (Vector Laboratories). Sections were visualized by fluorescent microscopy on a Ziess Axioplan 2 microscope.

### Western Blot Analysis

Western blot analysis was performed using 20 µg of protein per sample [28]. Control and mutant cerebellum were dissected from age matched P3, P7 and P11 mice and lysed in RIPA buffer (1X Tris buffered saline (TBS), 1% NP-40, 0.5% sodium deoxycholate, 0.1% SDS, 0.04% sodium azide, 1 mM PMSF). Samples were electrophoresed on 4–12% Tris-Bis NuPage gels (Invitrogen). Proteins were transferred onto PVDF membranes and western blot analysis was performed using the Odyssey Infrared Imaging System (LiCor Technologies) according to manufacturer's recommendations. Blots were incubated in Odyssey blocking solution for one hour at room temperature. Primary antibody: anti-GFAP at 1∶1000 (Chemicon), anti-CALB at (1∶2000) or anti-β-actin at 1∶1000 (Santa Cruz), was incubated in Odyssey blocking solution overnight at 4°C, then washed in PBS. The appropriate secondary antibody was incubated at 1∶10000 for one hour at room temperature (AlexaFluor680, Molecular Probes). Images were detected with the Odyssey system using the proper infrared channels and band intensity was measured using Odyssey v.2.1.12 software.

### Expression Analysis

Quantitative RT-PCR was used to determine the expression levels of *Eaat4* relative to *β*-*actin* in mutant and age-matched control animals. Total cerebellar RNA was isolated from three euthanized mutant and control littermates at P11 using Trizol reagent (Invitrogen) per manufacturer's instructions. The RNA was DNase treated with DNA-free (Ambion) to remove contaminating DNA, per manufacturer's instructions. First strand synthesis was performed using the RETROscript Kit (Ambion) on 2 µg of RNA template, per manufacturer's instructions. Reaction products were diluted 1 to 100 and 1 µl was used as template for amplification using the following 10 µl reaction mixture: 100 pmol each forward and reverse primer and 6 µl Sybr green PCR master mix (ABI). Forward and reverse primers for *Eaat4* and *β-actin* were chosen for each gene using Primer Express 3.0 (ABI). Quantitative RT-PCR was carried out on an ABI 7500 using the default cycling parameters. Reactions were performed in triplicate for the three separate RNA samples for wild-type and mutant. As a measure of amplification, the number of cycles to a manually set ΔR_n_ (dye fluorescence) threshold was determined as the ΔC_t_ value (ABI 7500 System Software). ΔC_t_ values were obtained for each sample, 9 total for each gene (3 triplicated replicates). Average ΔC_t_ for each gene was calculated for each dataset as well as standard deviation and standard error. Significance was determined by the t-test. The ΔΔC_t_ was derived by comparison to a control gene, β-actin, for each sample. Comparisons between mutant and wild-type control samples were made for fold change estimation using the comparative C_t_ method, 2^−ΔΔCt^, where ΔΔC_t_ = ΔC_t_test gene-ΔC_tβ_-actin, relative expression was calculated per 1000 molecules of β-actin, 1000/2^-ΔΔCt^.

## Results

### Early Onset Wasting and Cerebellar Signs in the *Inpp4a^wbl^* Mutant

**Figure 1:**
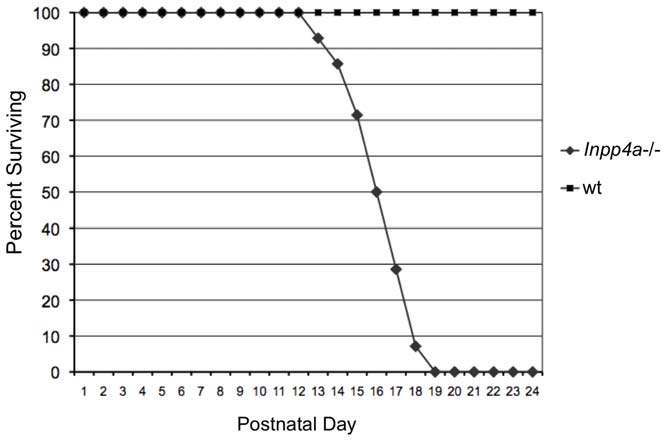
The *Inpp4a^wbl^* mutant phenotype causes pre-wean mortality. A Kaplan-Meier survival curve showing the lifespan of *Inpp4a^wbl^* mutants on the BALB/cJ background, n = 20, no heterozygous or wild-type pups were lost. Percent survival is plotted on the y-axis versus postnatal day on the x-axis.

The *Inpp4a^wbl^* mutant is characterized by an early onset recessive cerebellar ataxia. Consistent with many purely neurological mutants, there is a low incidence of *in utero* loss in mutants, with approximately 23% of live mice born from obligate carriers being affected (36/156). The *Inpp4a^wbl^* phenotype is predictable on the BALB/cJ inbred background and does not show a high degree of variability in terms of lifespan and vigor. At early perinatal timepoints mutant pups are indistinguishable from wild-type or heterozygous littermates. Subsequently, mutant mice fail to thrive with the average weight of a mutant at P9 being 3.1 g compared to an average of 6.9 g for wild-type (n = 3). A Kaplan-Meier survival curve shows that there is a precipitous mortality rate beginning at P13 (Figure 1). On average a mutant mouse survives until P16 (n = 20). In order to generate longer surviving mutants, in rare cases up to P24, a mixed genetic background was necessary. A heterozygous effect was observed in the activity of INPP4A toward PtdIns substrates in the mutant cerebellum [29]. To determine if the reduction in enzymatic capacity translated to a physical abnormality in coordination, heterozygous mice were tested by rotorod. Heterozygous mice had similar capabilities as wild-type during accelerating rotorod trials (n = 6) (data not shown). Mutant mice are not testable by rotorod.

### Purkinje Cells Are Lost at Early Perinatal Timepoints in the *Inpp4a^wbl^* Mutant

**Figure 2:**
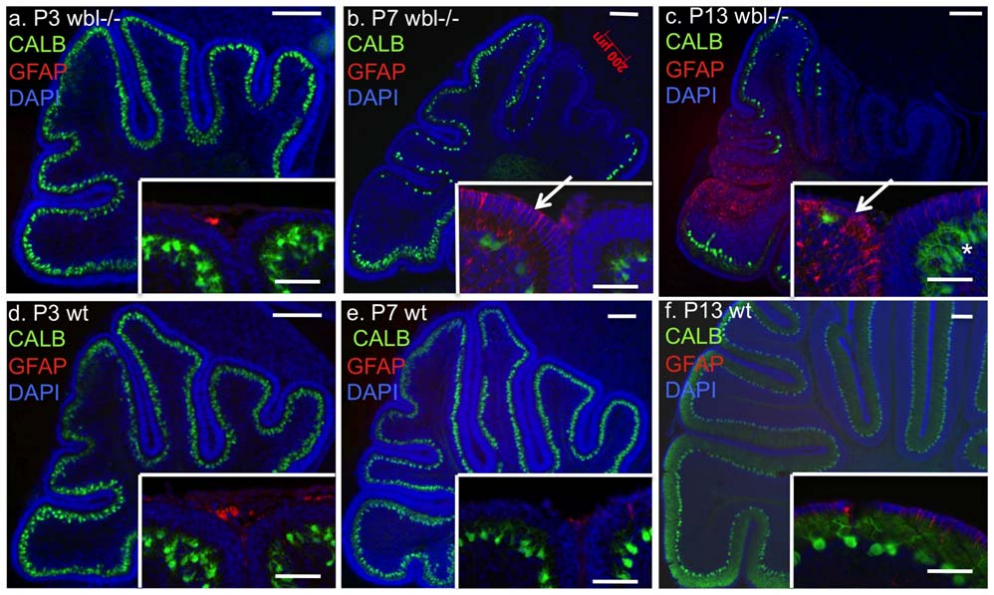
Rapid early onset Purkinje cell loss in *Inpp4a^wbl^* mutants. Sagittal sections at the midline of the cerebellum from postnatal day 3, 7 and 13 mutants (panels a,b,c) and matched wild-type littermate controls (panels d, e, f). All sections were matched using the superior cerebellar peduncle. Purkinje cells are labeled with anti-calbindin (green), reactive gliosis is indicated by anti-GFAP antibody (red) and sections are counterstained with DAPI (blue) (scale bar = 200 microns). High GFAP staining (red) indicative of reactive gliosis is seen restricted to certain lobes. Insets show neighboring folia with different degrees of gliosis and cell loss (arrows, scale bar = 50 microns) and surviving Purkinje cells in the mutant, which appear normal (asterisk).

**Figure 3:**
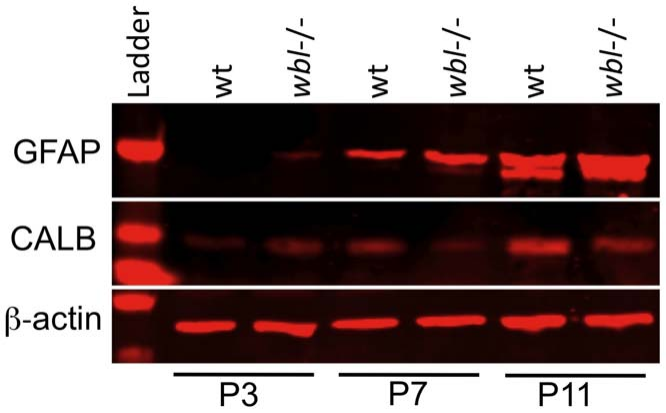
The mutant cerebellum has elevated levels of reactive gliosis at early perinatal timepoints. Protein extracts from wild-type (wt) and mutant (*wbl*−/−) cerebellum were analyzed by western blot using GFAP and Calbindin antibodies at P3, P7 and P11. β-actin is shown as a loading control.

In order to determine the progression of Purkinje cell loss, immunohistochemistry was performed on age matched mutant and control animals with anti-calbindin (CALB) antibody to label Purkinje cells and anti-GFAP antibody to identify reactive gliosis. Serial sections from several post-natal time points (n = 3 at each timepoint) were analyzed. Histological analysis showed that the course of degeneration has a patterned progression. In sagittal sections at P3 there is no difference between the wild-type and mutant cerebellum (Figure 2a,d). At P7, at midline sections, Purkinje cell loss and reactive gliosis can be seen restricted to specific folia (Figure 2b,e). Interestingly, neighboring lobes show drastic differences in their cell loss and levels of GFAP staining. The same pattern continues at P13, where degeneration is more widespread but areas of protected cells exist in certain folia (Figure 2c,f). In protected folia, Purkinje cells have a normal appearance (Figure 2c inset). Degeneration was not observed in heterozygous animals. Western blot analysis shows that GFAP is increased in mutants compared to wild-type, while CALB levels are decreased at P7 and P11 (Figure 3). At P7 and P11 a 50% and 141% increase in GFAP intensity is observed, respectively. Reactive gliosis is detected in P3 mutants prior to observable Purkinje cell loss. The increase in GFAP between ages in the wild-type is likely due to the increase in the number of cells as the cerebellum develops, and the apoptotic pruning in the granule cell layer that begins around P11.

### *Inpp4a* Is Expressed Uniformly in the Cerebellum

**Figure 4:**
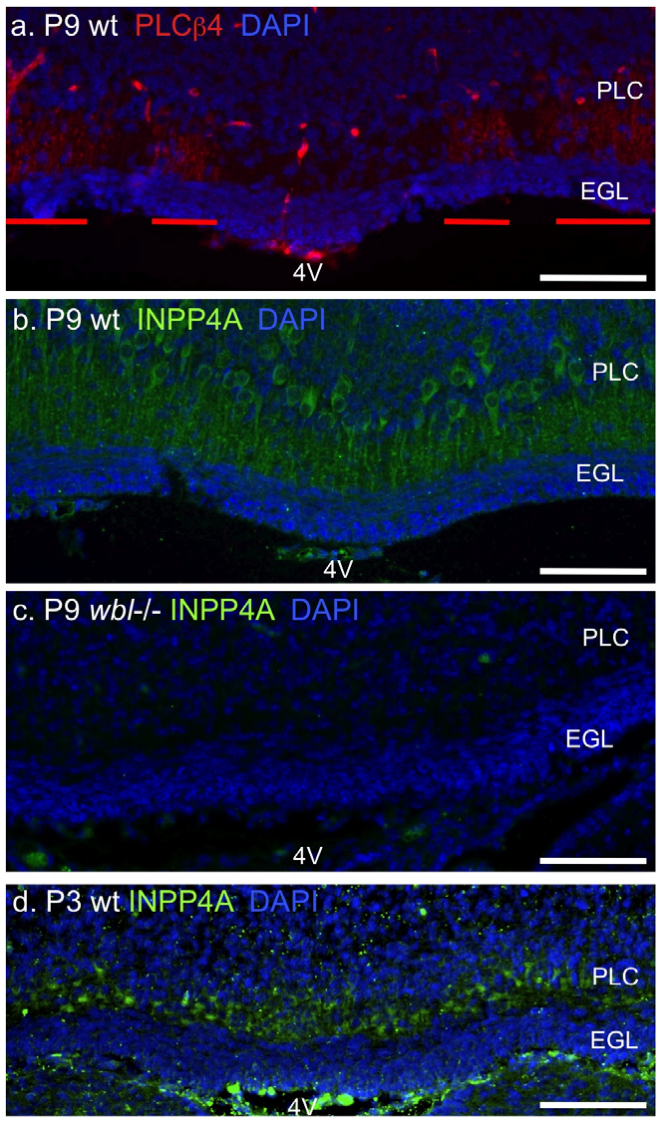
*Inpp4a* is expressed uniformly in Purkinje cells. Adjacent coronal sections from a wild-type P9 cerebellum labeled with anti-PLCβ4 (panel a), INPP4A (panel b) and mutant labeled with INPP4A (panel c). Coronal sections from wild-type P3 cerebellum show uniform *Inpp4a* labeling (panel d). Parasagittal striping of PLCβ4 is obvious at the midline superior to the 4^th^ ventricle (4V) in the P9 cerebellum(red bars). Sections are counterstained with DAPI, scale bar indicates 100 microns, PCL, Purkinje cell layer, EGL, external granule cell layer.

*Inpp4a* mRNA is highly expressed in Purkinje cells; however, it is unknown if *Inpp4a* expression is uniform across the cerebellum. Patterned expression of the mutant allele could be the basis for patterned neurodegeneration. We serially sectioned cerebella from P3 and P9 mutant, prior to- and when degeneration is obvious, and wild-type mice in the coronal plane and used immunohistochemistry to determine the expression pattern of INPP4A. The expression of INPP4A was uniform across the cerebellum and restricted to the Purkinje cell soma and their dendrites in the molecular layer (Figure 4b,d). PLCβ4 was used as a control in adjacent sections to show parasagittal striping at the midline superior to the fourth ventricle (Figure 4a). *Plcβ4* is one of the few genes know to stripe at early perinatal stages and maintain that pattern into adult stages. INPP4A was not detected in surviving Purkinje cells of the mutant mouse, demonstrating the specificity of the antibody (Figure 4c).

### Abnormal Elimination of Climbing Fibers in the *Inpp4a^wbl^* Cerebellum

**Figure 5:**
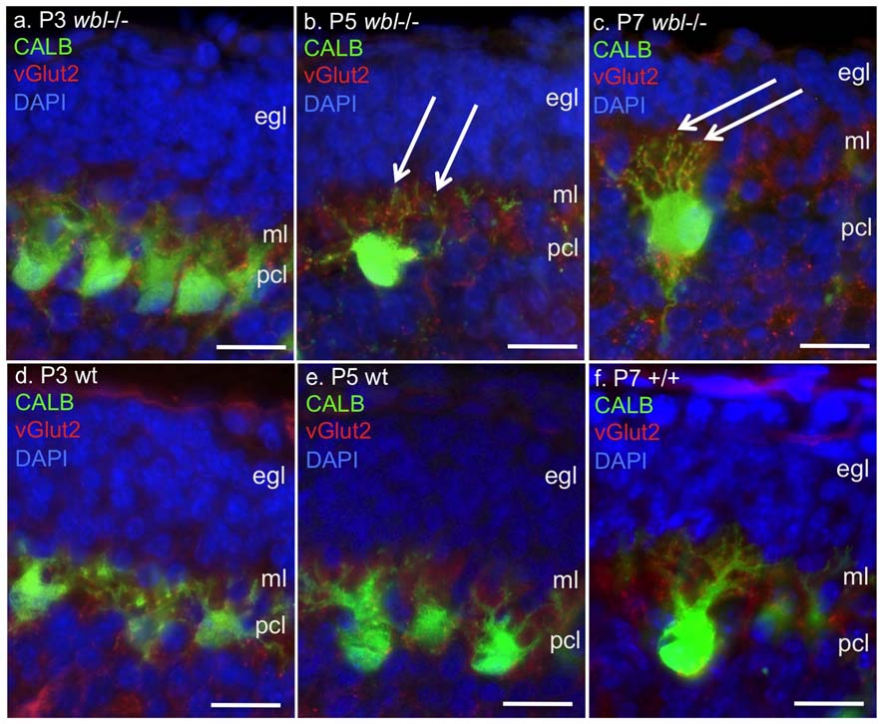
Purkinje cell loss coincides with the appearance of climbing fibers in the developing molecular layer. Panels d, e, f show climbing fibers labeled by anti-vGlut2 (red) and Purkinje cells labeled by anti-calbindin (green) in wild type controls at P3, P5 and P7. Climbing fibers can be seen to extend to the limit of the developing molecular layer (ml) inferior to the external granule cell layer (egl). In age-matched mutant littermates (panels a,b,c) Purkinje cells (pcl) have an unusual appearance beginning at P5, multiple thin dendrites are seen with a prominent staining rounded soma (arrow). Scale bar indicates 25 microns.

**Figure 6:**
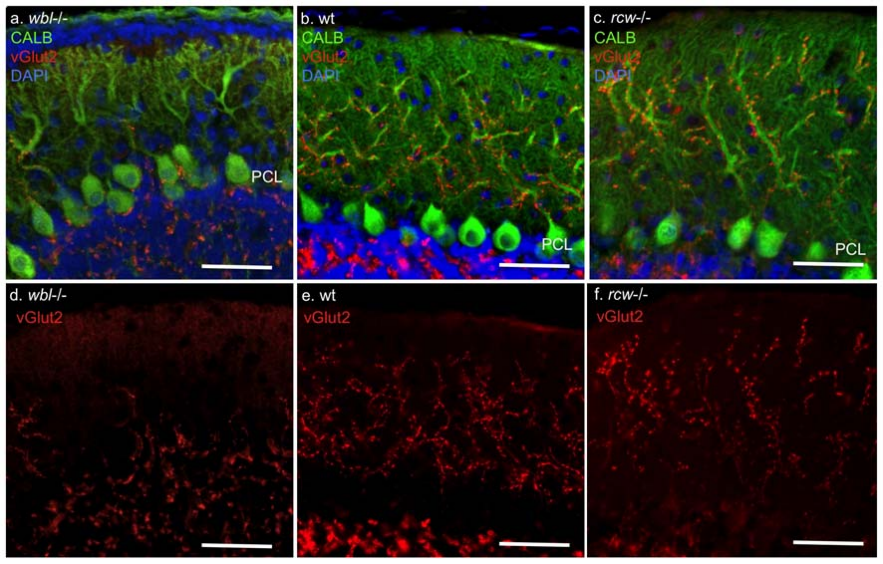
Climbing fibers are reduced in late stage *Inpp4a^wbl^* mutants. Panel a shows the *Inpp4a^wbl^* mutant at age P24, Purkinje cells are labeled with anti-calbindin (green) while climbing fibers are labeled by anti-vGlut2 (red). Panels b and c show wild-type control and *Grm1^rcw^* mutant for comparison. Panels d, e and f show climbing fibers in red from the unmerged image. Scale bar indicates 100 microns, (PCL), Purkinje cell layer.

Climbing fibers from the inferior olive stimulate glutamate receptors on Purkinje cells and therefore likely initiate the first usage of the inositol second messenger system. In order to determine if climbing fiber synaptogenesis is a possible trigger for Purkinje cell loss, mutant and wild-type littermate cerebellum were immunostained with antibodies to CALB to label Purkinje cells and vesicular glutamate transporter 2 (VGLUT2) to label climbing fibers. At P3, prior to Purkinje cell loss, climbing fibers can be seen extending into the developing molecular layer (Figure 5a,d). At P5 and P7 in the mutant, Purkinje cells appear distressed with rounded soma and multiple atrophic dendrites extending into the developing molecular layer (Figure 5b,c). In some cases four to five dendrites can be seen extending off the soma. At P7 an average of 8.4 distressed Purkinje cells were identified in GFAP positive regions (average linear distance counted was 270 µm, n = 8). In later stage mutants, surviving Purkinje cells have properly remodeled their dendrites; however, climbing fibers appear to have been excessively eliminated in the *Inpp4a^wbl^* mutant (Figure 6a,d). Compared to wild-type and mutant *Grm1^rcw^*, which have an abnormal excess of climbing fibers (Figure 6c), the molecular layer in the *Inpp4a^wbl^* mutant is relatively devoid of climbing fibers. In an average 225 µm linear area from late stage mutants, approximately 7 of 10 Purkinje cells had completely eliminated all climbing fibers (n = 14). Varicosities were observed in approximately 30% of the mutant Purkinje cell dendrites (n = 12).

### Protected Purkinje Cells Are Located in EAAT4 Positive Parasagittal Stripes

**Figure 7:**
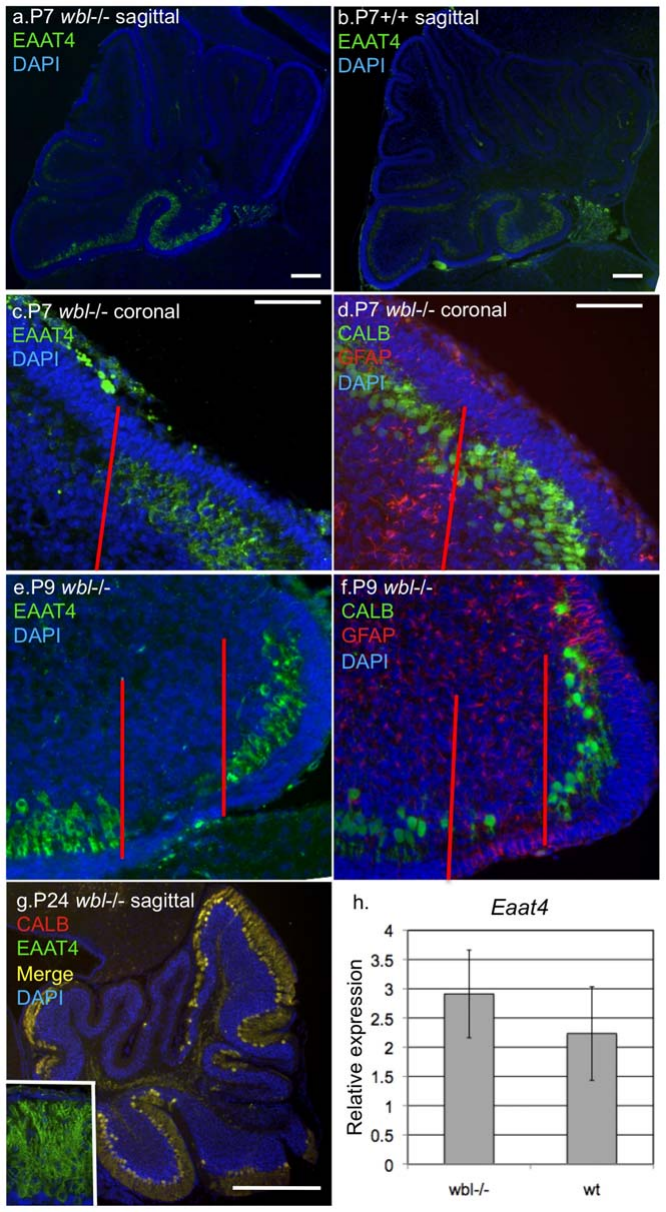
EAAT4 expressing Purkinje cells are protected in early stage *Inpp4a^wbl^* mutants. At P7 in midline sagittal sections EAAT4 is restricted to the most caudal lobes in mutant and wild-type (panels a, b). In adjacent coronal sections at P7 a parasagittal stripe of EAAT4 expression is observed, while all Purkinje cells are observed in the neighboring section when stained with calbindin (panels c, d). Reactive gliosis is seen in the EAAT4 negative stripe prior to Purkinje cell loss (panel d left of red line). At P9 most EAAT4 negative Purkinje cells have degenerated leaving a few abnormal Purkinje cells and significant gliosis (panels e, f). At P24 all remaining Purkinje cells are those that express EAAT4 (panel g, inset shows single labeled EAAT4). Quantitative real-time PCR shows that *Eaat4* expression is preserved in mutant brains (panel h).

Immunohistochemistry and quantitative gene expression analysis were used to determine if persistence of Purkinje cells was correlated to the expression of a protective gene. In sagittal sections at P7 both mutant and wild-type show that EAAT4 is localized to the most caudal lobes of the developing cerebellum, a region where Purkinje cells are protected (Figure 7a,b). Serial coronal sections were used to define the pattern of Purkinje cell loss respective to *Eaat4* patterned parasagittal gene expression. Mutant cerebella were immunostained with anti-EAAT4 and adjacent sections were immunostained with anti-CALB and GFAP. At P7 CALB is uniformly expressed while in the adjacent section a clear division of EAAT4 negative and EAAT4 positive regions (Figure 7c). Reactive gliosis is seen in the mutant EAAT4 negative stripe (Figure 7d). At P9 only a few EAAT4 negative cells are identifiable and they appear distressed and are surrounded by reactive gliosis (Figure 7e,f). Relatively little gliosis is seen in EAAT4 positive areas. Cerebellar sections from a late stage mutant were double immunostained with CALB and EAAT4 and showed that all surviving Purkinje cells express EAAT4 (Figure 7g). Quantitative RT-PCR was performed on P11 stage mutants and independently confirmed that EAAT4 expression was maintained at wild-type levels in the mutant cerebellum (Figure 7h).

## Discussion

In this report we have identified several unique features of the *Inpp4a^wbl^* phenotype that are suggestive of the molecular mechanism of degeneration. In the *Inpp4a^wbl^* mutant, Purkinje cells are lost early and rapidly, coinciding to the developmental stage when climbing fiber connections are being made. Purkinje cells that are lost show a striking morphology. Multiple atrophic dendrites are seen extending from a rounded soma and reactive gliosis is detectable prior to appreciable Purkinje cell degeneration. This would correspond to the first major usage of the inositol second messenger system. Consistent with the genetic defect there are no histological abnormalities prior to the initial use of the pathway involving the disease gene. In striking contrast to the precipitous loss of Purkinje cells in some regions, other areas of the cerebellum have normal appearing Purkinje cells. This degenerative pattern is not due to the differential expression of the disease gene, as we have shown that *Inpp4a* expression is not compartmentalized. However, the pattern of protection coincides with *Eaat4* expression. The early onset and patterned nature of Purkinje cell death makes this mutant an efficient model for neuroprotective studies.

The inositol metabolism pathway is involved in diverse functions in the cell [30]. The deficiency of INPP4A is known to cause defects in the metabolism of insoluble PtdIns substrates. A reduction in the 4-phosphatase activity toward PtdIns(3,4)P_2_ was shown in extracts from mutant cerebella compared to wild-type and this substrate was also found to be at higher levels in cultured astrocytes [29]. The enzyme is known to colocalize with early endosomes and dilated early endosomes are observed in the mutant [31]. Further, knockdown of INPP4A was demonstrated to reduce the internalization of transferrin [29]. While a heterozygous effect is observed for enzyme activity levels, there is no physical abnormality observed in heterozygotes. However, it is possible that accelerating rotorod analysis is simply not sensitive enough to identify minor differences in motor coordination.

It is unclear whether the disruption of the PtdIns pathway contributes to neurodegeneration. Mutants that affect PtdIns are not normally associated with a degenerative phenotype, and vesicle transport can be completely disrupted without neurodegeneration. It is more likely that degeneration results from the disruption of soluble substrates, absence of *Inpp4a* would cause a block in the metabolism of inositol 1,4,5-triphosphate (IP_3_) to inositol (Ins). IP_3_ is involved in the regulation of Ca^2+^ release from intercellular stores [32], [33]. Due to the important role of *Inpp4a* in inositol metabolism, one likely disease mechanism is that the regulation of intracellular Ca^2+^ is disrupted leading to cytotoxic stress. Large increases in intracellular Ca^2+^ activate self-destructive enzymes, such as calpains and caspases, increase the production of free radicals, and impair the function of organelles [34], [35]. In Purkinje cells this pathway is triggered by glutamate binding to glutamate receptors, such as GRM1, and attenuated by glutamate transporters, such as EAAT4.

It is known that climbing fibers activate the GRM1 on the Purkinje cell dendrite. The climbing fiber-Purkinje cell connection is one of the most powerful synaptic connections between two neurons. The normal process of climbing fiber elimination is abnormal in the mutant. Unlike the *Grm1* mutant, which has excessive climbing fibers [36], the *Inpp4a^wbl^* mutant climbing fibers develop normally until P15 when elimination begins. Mutant climbing fibers appear to be over-eliminated. This could be protective of Purkinje cells in late stage mutants, as it has been shown that the transection of the climbing fiber inputs from the olive is protective in ischemia [25]. The process of climbing fiber elimination is not well understood, and the *Inpp4a* mutant appears unique in this defect. This is probably a reflection that these genes are on opposite sides of the same pathway, *Grm1* being involved in the production of IP_3_, *Inpp4a* being responsible for the breakdown of IP_3_.

In Purkinje cells *Eaat4* is expressed in parasagittal stripes and localized to the postsynaptic spines [18], [37]. It is activated by climbing fibers and is the most active neuronal glutamate transporter for Purkinje cells [18]. *Eaat4* functions to remove glutamate from the synapse and controls the activation of metabotropic glutamate receptor [39], [40]. In order for GRM1 to become activated in areas of the cerebellum with high *Eaat4* expression, extremely high levels of glutamate must be present [41]. In the *Inpp4a^wbl^* mutant, it appears that *Eaat4* is protective of Purkinje cell. Purkinje cells that are lost early are located in *Eaat4* negative stripes and all late stage surviving Purkinje cell express *Eaat4*. Correspondingly, expression of *Eaat4* is preserved in the mutant relative to wild type by quantitative RT-PCR. While this could be as coincidental as the colocalization of any other striped gene and surviving Purkinje cells, the fact that the two genes act in the same pathway suggest that *Eaat4* is protective.

Parasagittal *Eaat4* expressing stripes of Purkinje cells are preserved in ischemic models of cell death [25], [42]. Various stripping patterns have been noted in mutants with degenerative Purkinje cells; however, direct causal relationships between compartmentalized genes and degeneration have not been established. Direct evidence would have to include knockdown or expression of *Eaat4* across its normal boundaries. Given the molecular relationship between *Eaat4* and *Inpp4a* and the phenotype of the mutant mouse, this mutant model would be an ideal system to establish causality.

These data presented in this manuscript suggest a possible model for cell death and protection in the *Inpp4a^wbl^* cerebellum. Glutamate released from the climbing fiber causes the production of IP_3_ in Purkinje cells, the mutant allele causes a disruption in the breakdown of IP_3_, resulting in excessive Ca^2+^ release into the cytoplasm. In areas with high *Eaat4* expression, glutamate release from the climbing fibers is rapidly cleared from the synapse and does not stimulate the production of IP_3_, thus protecting the neuron from it an inherent defect in processing that substrate.
